# Persistent directional cell migration requires ion transport proteins as direction sensors and membrane potential differences in order to maintain directedness

**DOI:** 10.1186/1471-2121-12-4

**Published:** 2011-01-22

**Authors:** Nurdan Özkucur, Srikanth Perike, Priyanka Sharma, Richard HW Funk

**Affiliations:** 1 Department of Anatomy, Medical Faculty Carl Gustav Carus, Technical University of Dresden, Dresden, Germany

## Abstract

**Background:**

Ion transport proteins generate small electric fields that can induce directional cell motility; however, little is known about their mechanisms that lead to directedness. We investigated Na, K-ATPase (NaKA) and Na+/H+ exchanger isoforms (NHE1 and 3) in SaOS-2 and Calvarial osteoblasts, which present anode- and cathode- directed motility, during electrotaxis.

**Results:**

Significant colocalizations of NaKA with vinculin and pNHE3 with ß-actin were observed to occur at the leading edges of cells. The directedness were attenuated when NaKA or NHE3 was inhibited, confirming their implication in directional sensing. Depending on the perceived direction, a divergent regulation in PIP2 levels as a function of NHE3 and NaKA levels was observed, suggesting that PIP2 may act as a spatiotemporal regulator of the cell membrane during electrotaxis. Moreover, at the same places where pNHE3 accumulates, bubble-shaped H^+ ^clouds were observed, suggesting a physio-mechanical role for NHE3. The cell membrane becomes hyperpolarized at the front and depolarized at the back, which confirms NaKA activity at the leading edge.

**Conclusion:**

We suggest a novel role for both NaKA and NHE3 that extends beyond ion translocation and conclude that they can act as directional sensors and V_mem _as a regulatory cue which maintain the persistent direction in electrotaxis.

## Background

Directional cell motility plays an essential role in many biological processes, such as tissue formation/regeneration, wound healing, or tumor metastasis, and can be induced by both endogenously occurring and externally applied electric fields (EF). Persistent directionality requires precise, dynamic and regularly repeated cycles of interactions between cytoskeleton proteins, cell membranes, and the extracellular matrix so as to promote de novo protrusions at the leading edge of migrating cells, which distinguishes this process from random cell movement [1]. Focal adhesion complexes at the cell membrane-matrix interface allow for forward cell locomotion through rapid protein turnover, which results in a dynamic connection/disconnection to the matrix as cells move in a preferred direction. Thus, proteins that are preferentially located at these sites during directional movement may play a significant role in direction sensing. Previous works, which have investigated different cell types, have observed that many proteins, lipids, and organelles redistribute because cells are polarized during electrotaxis [2-4]; however, the molecules and mechanisms that enable cells to perceive direction during electrotactic, persistent motility have yet to be investigated.

Apart from the many studies that have investigated the cytoskeleton proteins that modulate cellular migration machinery, recent studies have focused on membrane ion transporters and their interactions with cytoskeleton proteins [5-7], specifically in their potential roles in cell motility. The emerging roles of membrane ion transport proteins in the control of electrotaxis and in directionally persistent cell migration have first been reported for the voltage-gated Na^+ ^channel in rat prostate cancer cells [8], followed by other studies concerning PKD2 cation channels in directional sperm movement [9], aquaporins in astroglial cell migration [10], potassium channels in the invasiveness of embryonic stem cells [11], TRP channels in embryonic lung fibroblast motility [12], and a recent report from our own work concerning voltage-gated calcium channels in the electrotaxis of osteoblast cells [13].

Aside from ion translocation, several recent studies have reported on the roles of both NHE and NaKA in cytoskeletal remodeling, cell polarity, and lamellipodia formation [14-18]. When taken together, especially in regard to their primary functions as pH or cell volume regulators and cellular migration-specific membrane potential state modulators, NHE and NaKA can provide new insights into the understanding of both the physiological and the mechanical regulation of directional sensing in cells.

Herein, we suggest that NaKA and NHE3 can act as directional sensors in EF-induced directional cell motility via a mechanism that involves PIP2 as a potential mediator and the cell membrane potential (V_mem_) as a regulatory cue. Using SaOS-2 and Calvarial osteoblasts, which represent anode- and cathode- directed motility, respectively, we show that 1) active NHE3 is concentrated in membrane protrusions that are accompanied by proton fluxes (pH_i_) at the leading edge of the cellular migration, especially in cathode-directed cells, and its activity is required for the perception of direction; 2) NHE1 is homogenously localized throughout the surface membrane and is involved in directional migration; 3) V_mem_, as a result of NaKA activity, has a regulatory function that maintains the persistent directionality by modulating the spatiotemporal changes between the leading edge (hyperpolarized) and the rear end (depolarized) in directionally migrating cells.

## Results

### NHE3 and NaKA colocalize with ß-actin and vinculin on the leading-edge of the cell

**Figure 1:**
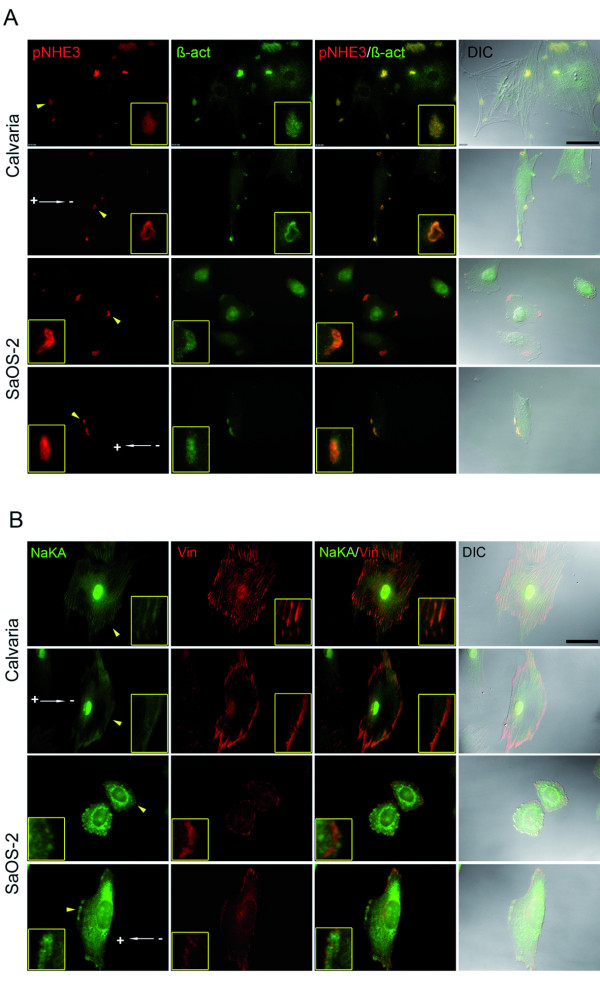
**The localization of pNHE3 and of NaKA in EF-directed- versus random cellular movement**. The colocalization of pNHE3 with ß-actin (A) and of NaKA with vinculin (B) on the leading edge of a cell in both cathode (Calvaria)- and anode (SaOS-2)-directed cells. First and third row: randomly migrating control cells. Second and fourth row: polarized cells that persist in a direction under an applied EF. White arrows with "+" and "-" poles indicate the migration direction under an applied EF. Yellow arrows indicate the magnified view and correspond to the entire row. Bar: 50 μm.

**Figure 2:**
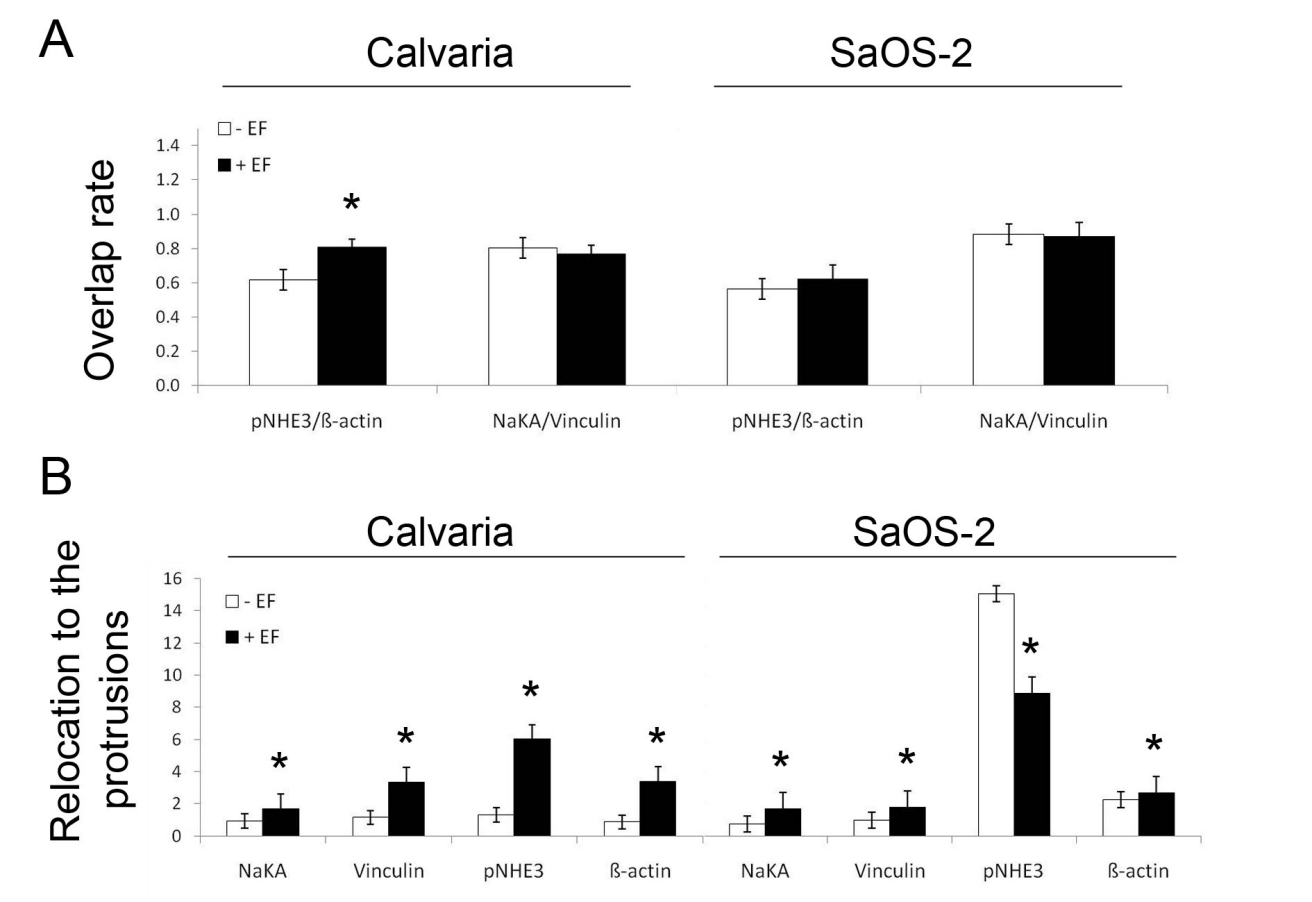
**Graphics showing the colocalization rate and relocation of proteins during electrotaxis.** The colocalization rates of pNHE3/ß-actin and of NaKA/vinculin with and without EF exposure in cathode (Calvaria)- and anode (SaOS-2)-directed cells (A). The EF-induced relocation of pNHE3, NaKA, ß-actin, and of vinculin to or from the leading edge of a cell during cathode (Calvaria)- and anode (SaOS-2)-directed motility (B). Error bars represent the SEM for 16-31 cells from three separate experiments (*P < 0.05 compared to the control -wothout EF-).

Phosphorylated NHE3 (pNHE3) was observed to specifically accumulate at the membrane protrusions of polarized (EF) and non-polarized (control) cells. Interestingly, pNHE3 only appeared at the same places where ß-actin accumulates, which represents significant colocalization at cell leading-protrusions in both anode (SaOS-2)- and cathode (Calvaria)-directed cells (Figure 1A). The colocalization rate of pNHE3/ß-actin was observed to increase by 30.4% and by 9.8%in cathode- and anode- directed cells, respectively, during electrotaxis (Figure 2A). The total NHE3 was evenly distributed on the cell membrane with less significant accumulations in the protrusions (Additional file 1). NHE1 exhibited a homogenous distribution throughout the cell membrane, regardless of cell polarization or migration direction (Additional file 2).

Unlike NHE3, the total NaKA was observed to specifically accumulate at the membrane protrusions of polarized and non-polarized cells. The pattern of accumulated NaKA, which was especially striking in calvaria cells, resembled that of the focal adhesion marker protein, vinculin, which directed us to further investigate whether NaKA and vinculin are colocalized in cell membrane protrusions. Indeed, significant colocalizations of NaKA with vinculin were observed at the cell periphery of non-polarized cells and at the leading edge of polarized cells during both cathodal (Calvaria) and anodal (SaOS-2) cell motility. Additionally, NaKA was localized also at the cell nuclei (Figure 1B). Very slight decreases of 0.1% and 1.4% in cathode- and anode-directed cells, respectively, in the colocalization rate of NaKA/vinculin were observed during electrotaxis (Figure 2A). Phosphorylated NaKA was homogenously distributed on the cell membrane during directed motility (Additional file 3).

### EF induces the relocation of proteins to the cell leading-protrusions

In order to define the degree of protein relocation to the leading edge during directed cell movement, we plotted the distribution of each protein as a ratio of the mean fluorescence intensity in protrusions to that of the cytoplasm in polarized and in non-polarized cells. The fluorescence intensity ratios for the total NaKA, vinculin, and ß-actin were greater in polarized cells than in non-polarized cells, which reveal that these proteins were relocated from the cytoplasm to the membrane protrusions during directional cell movement. In both cathode- and anode-directed cells, relocation rates of NaKA from cytoplasm to cell leading edge were enhanced by 83% and 127%, respectively, during electrotaxis. In cathode-directed cells, relocation rate of vinculin and ß-actin to the leading edge of the cell was enhanced by 300 and 400%, respectively, whereas in anode-directed cells, vinculin and ß-actin were relocated by only 82% and 19%, respectively; however, the relocation of pNHE3 to the leading edge was enhanced by 400% in cathode (Calvaria)-directed cells, whereas it was diminished by 41% in anode (SaOS-2)-directed cells (Figure 2B). PIP2 was observed to be located along the cell periphery during both cathodal and anodal movement; however, it was also located along the cell periphery in randomly migrating cells (Additional file 4).

### Cellular directedness is regulated by the activity of NHE3, NHE1 and NaKA

**Figure 3:**
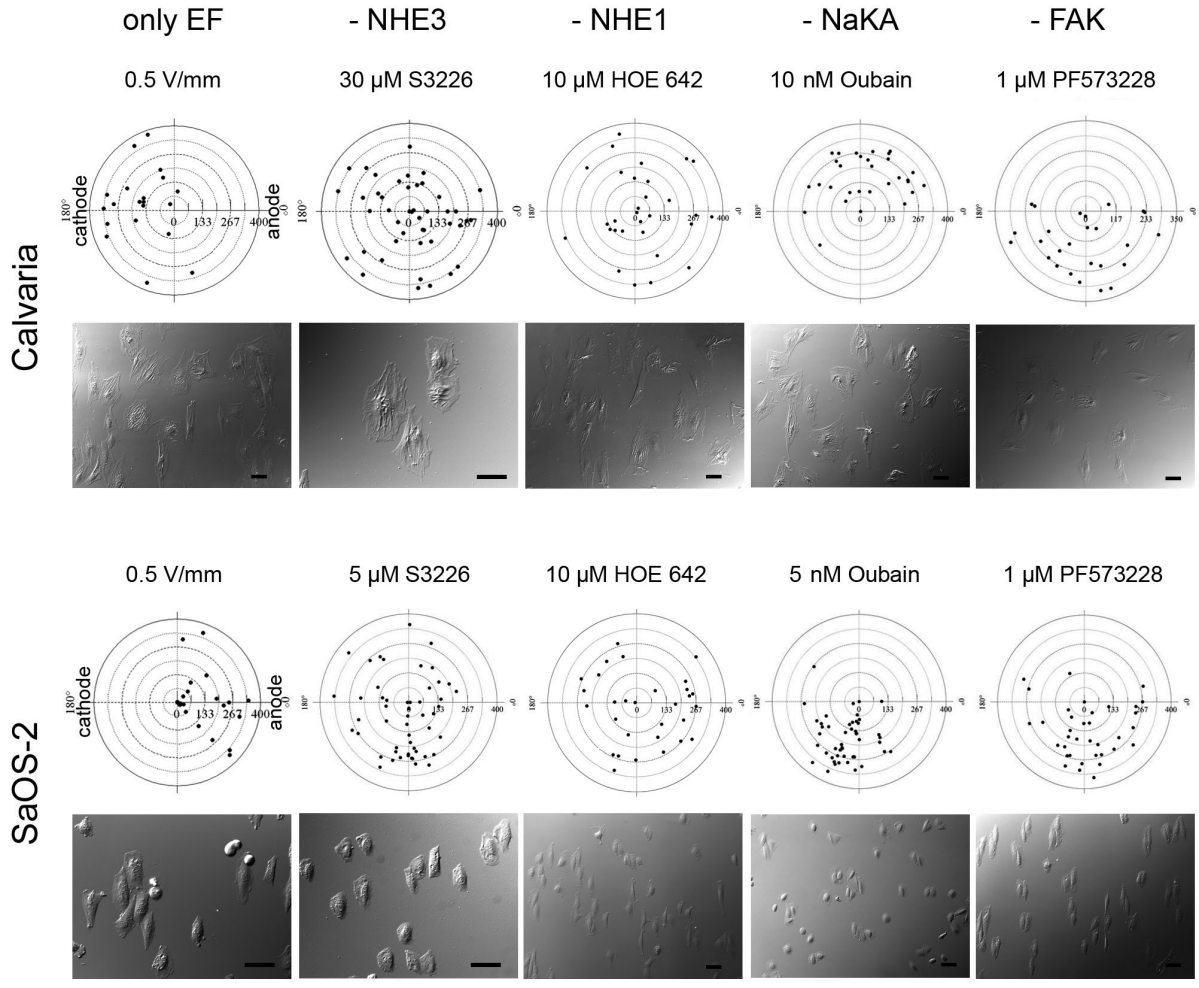
**Polar diagrams representing the directedness of cathode (Calvaria)- and anode (SaOS-2)-directed cells in the presence of an applied EF or an EF in combination with the NHE3-, NHE1-, or NaKA-specific inhibitors, S3226, HOE 642, or oubain, respectively**. FAK inhibition was used as a positive control. Each diagram represents the data from 25-45 cells from three to five individual experiments. DIC images showing the cell shape, morphology, and orientation in the presence of EF only or with inhibitors. Bars: 50 μm.

EF-induced cellular directedness was attenuated when NHE3, NHE1, or NaKA activity was suppressed via the use of the specific inhibitors, S3226, HOE 642 or Oubain, respectively, in both cathode- and anhode-directed movement. Cells that were treated with the FAK inhibitor, PF573228, were used as a positive control. Although the cells exhibited an elongated shape in the applied EF as normal, they failed to form a polarized shape, which is characterized by a well-defined leading edge and rear end, when NHE3, NHE1, or NaKA was inhibited (Figure 3).

**Figure 4:**
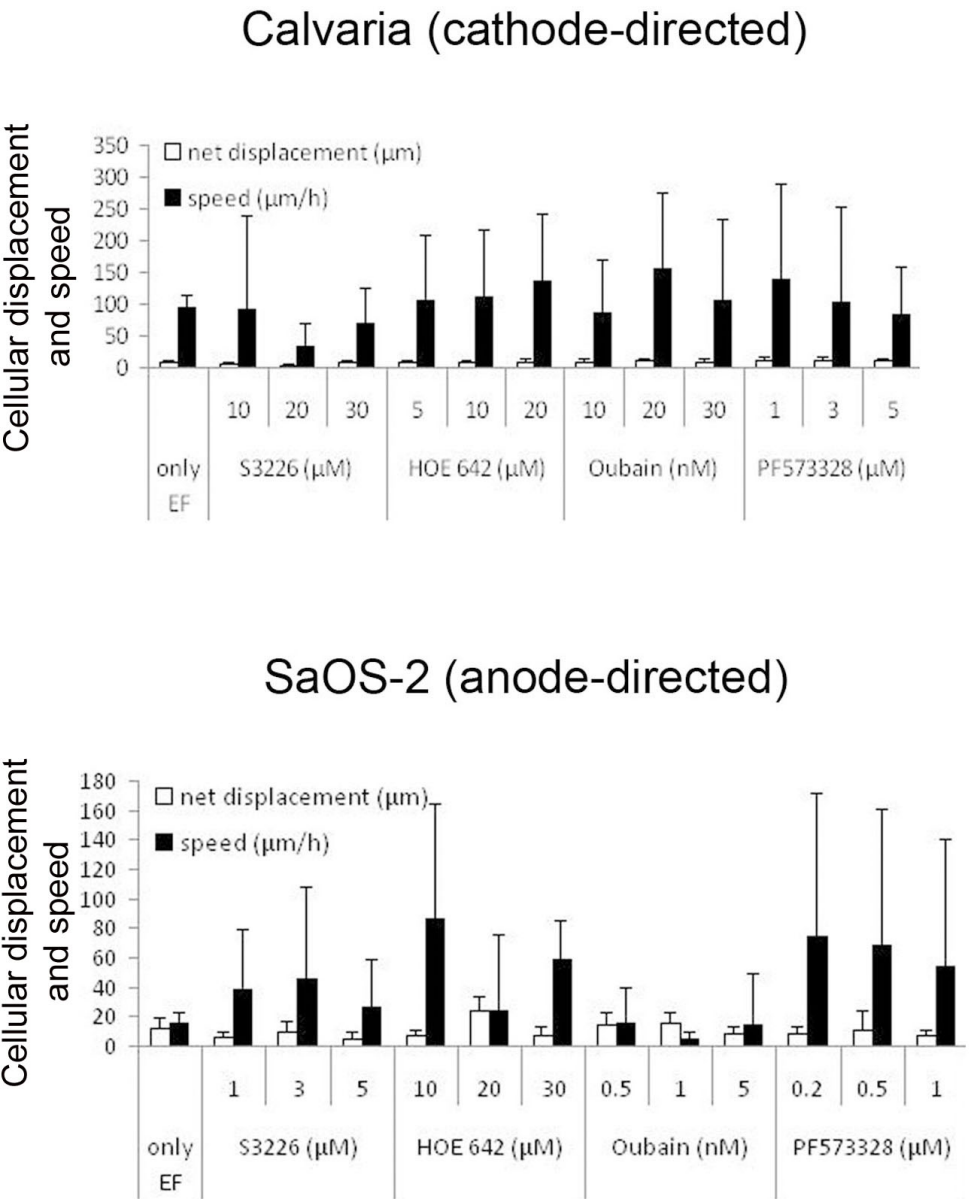
**The speed (μm/h) and net linear displacement (μm) of cathode (Calvaria)- or anode (SaOS-2)-directed cells in the presence of an applied EF or, an EF in combination with the NHE3-, NHE1-, or NaKA-specific inhibitors, S3226, HOE 642, or Oubain, respectively**. Error bars represent the SEM for 25-45 cells from three to five separate experiments.

The inhibition of NHE3, NHE1, or NaKA slightly affected (a maximum of 50%) the net displacement of both cathode- or anode- directed cells. In contrast, especially in anode-directed cells, inhibition dramatically affected the cellular speed. SaOS-2 cells exhibited a 300% increased speed when NHE3 was inhibited (3 μM S3226) and a 500% increased speed when NHE1 was inhibited (using 10 μM HOE 642). In cathode-directed cells, the pharmacological inhibition of NHE3 and NHE1 resulted in an average increase of only 21.5% and 25.3%, respectively. The inhibition of NaKA divergently affected the cellular speed in cathode- and in anode- directed cells. The cellular speed was increased by 67% (using 20 nM Oubain) in cathode- and decreased by 71% (using 1 nM Oubain) in anode-directed cells when NaKA was inhibited. The inhibition of FAK, which served as a positive control in this study, caused a 500% (using 0.2 μM PF573328) increase in cellular speed in anode-directed cells, whereas the speed of cathode-directed cells increased only by 48% (using 1 μM PF573328) (Figure 4).

### The intracellular levels of pNHE3 which are associated with PIP2, are divergently regulated depending on the perceived direction during electrotaxis

**Figure 5:**
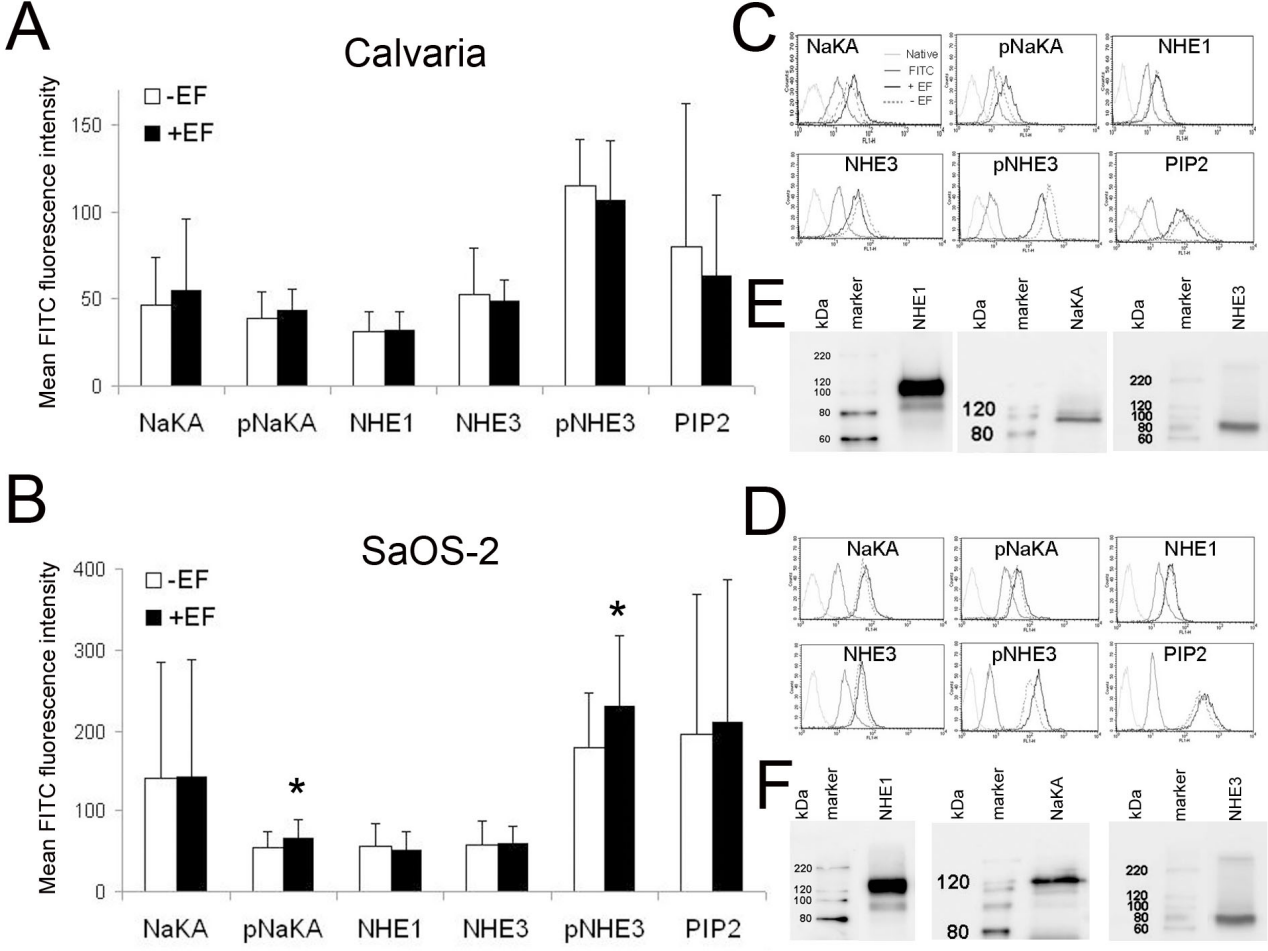
**Changes in the protein amounts during electrotaxis.** Intracellular levels of NaKA (total + phosphorylated), NHE1, NHE3 (total + phosphorylated) and PIP2 in cathodally (Calvaria) and in anodally (SaOS-2) migrating cells (A, B). Error bars represent the SEM for 11-16 separate measurements (*P = 0.01). FACS histograms that represent original measurements of NaKA (total + phosphorylated), NHE1, NHE3 (total + phosphorylated), and PIP2 from cathodally (Calvaria) and from anodally (SaOS-2) migrating cells (C, D). Images from the Western blots which show the osteoblastic isoforms of the NaKA (whole cell lysate, 110 kDa), NHE1 (whole cell lysate, 92 kDa), and NHE3 (immunprecipitate, 85 kDa) used in this study (E, F).

The intracellular levels of both total and phosphorylated NHE3 decreased by 7%, which was also associated with a 21% decrease in PIP2 levels, in cathodally migrating cells (Calvaria). In contrast, the total and phosphorylated NaKA levels were elevated by 17% and by 12%, respectively, in cathode-directed cells. The NHE1 level was not affected during cathodal electrotaxis (Figures 5A and 5C). In anodally migrating cells (SaOS-2), the intracellular level of phosphorylated NHE3 increased by 29% whereas the total NHE3 level was not affected. Furthermore, the PIP2 level in anode-directed cells was also increased by 7%. Similar to what was observed for NaKA levels in cathode-directed cells, phosphorylated NaKA levels also increased by 23% in anode-directed cells, but without a change to the total NaKA levels. The NHE1 level decreased by 7% during anodal electrotaxis (Figures 5B and 5D).

### The cell membrane is relatively hyperpolarized at the leading edge versus the rear end during directed migration

Regardless of migration direction, different membrane potential at two ends of the cell was observed, that is, in both cathode- and anode-directed cells; the cell membrane exhibited a relatively hyperpolarized state on the leading edge in comparison to the rear end during electrotaxis. Taking advantage of the V_mem _reporter dye, DiBAC_4_(3), which is taken up by cells through the depolarized membrane, we monitored the spatiotemporal differences between the front and back ends of polarized cells during electrotaxis. In both cathode- and anode-directed cells, we observed that DiBAC_4_(3) was taken up first from the rear end, whereas there was no specific direction of dye uptake in randomly migrating control cells (no EF). V_mem_ kinetics collected from the selected region of interests (R) during the time-lapse recording of the cells loaded with DiBAC_4_(3) confirms that the leading edge of the cells has lower fluorescence intensity profiles (hyperpolarized) than the rear end (depolarized). However, it is also important to notice that the limitations of the technique such as fluorescence signal relevant to the morphological differences should be kept in mind when using potentiometric-, single excitation dyes.

**Figure 6:**
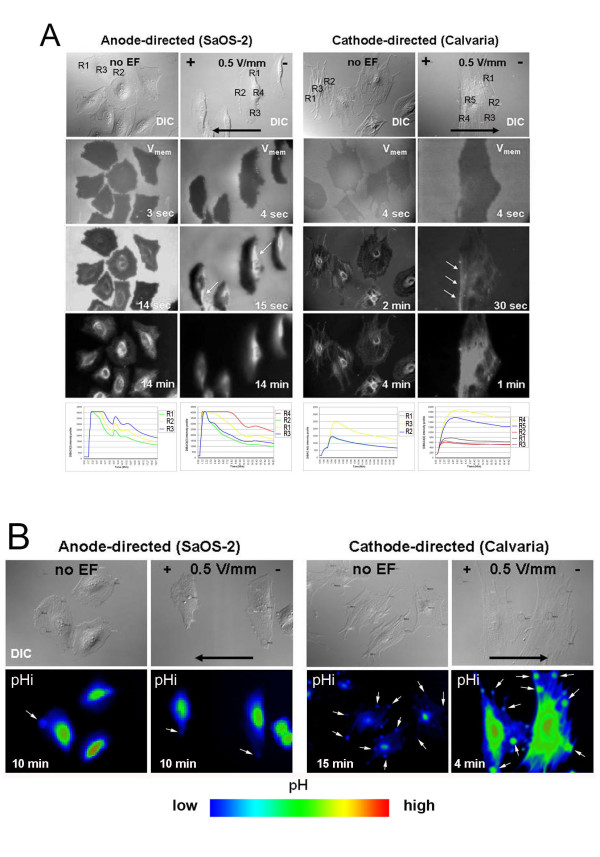
**Spatiotemporal differences in V_mem _between the leading edge and the rear end in cathode (Calvaria)- or anode (SaOS-2)-directed cells**. Fluorescence images showing the uptake of V_mem _reporter dye DiBAC_4_(3) at different time points after addition to the cells. White arrows in the second row point to the depolarized cell membrane, wherein the anionic dye first enters from sites that have a decreased number of negative charges (depolarized) at the rear end in both anode (left)- and cathode (right)-directed cells (A). pH bubbles (white arrows), especially recognizable in cathode-directed cells (right column), can be observed at the termini of membrane protrusions along the cell periphery in randomly migrating, nonpolarized cells and at the leading-edge of directed, polarized cells. False colored (LUT) images generated from ratiometric images showing cells loaded with BCECF-AM, a pH reporter, ratiometric dye (B). In both (A) and (B), the first row shows DIC images of the cells during random (no EF) and directed (0.5 V/mm) migration. Black arrows in the DIC images indicate the direction of migration.

All taken together, this observation reveals that the cell membrane had a relatively depolarized state at the rear end versus the leading edge or similarly, a relatively hyperpolarized state at the leading edge versus the rear end (Figure 6A, Additional files 5 and 6).

### pH_i_ is elevated in the membrane protrusions at the leading-edge of cathode-directed cells but not in anode-directed cells

Cathodally migrating cells, which have a well-defined polarized and elongated morphology, were observed to have bubble-shaped outward proton fluxes that are specifically located at the termini of membrane protrusions on their leading edge during electrotaxis. False colored (LUT) images shown in figure 6B represent the spatiotemporal differences in the BCECF-AM intensity profile, indicating the changes in pH_i_. These sites were likely to be the same regions where pNHE3 had specifically accumulated on the leading edge (Figure 6B, Additional file 7). Randomly migrating control cells (no EF) also exhibited pH bubbles, but they were located non-specifically along the cell periphery. Conversely, there was either one or no bubble per cell in anode-directed cells (SaOS-2) and in their controls (SaOS-2, no EF) during electrotaxis.

## Discussion

This study demonstrates that NaKA, NHE3, and NHE1 are involved in cellular directedness during electrotaxis, which imply that V_mem _is a spatiotemporal regulator between the leading edge and rear end. We suggest that NHE3 might be a cathode-specific sensor protein because there was an increased relocation of active NHE3 to the membrane protrusions, which was accompanied by an elevated pH_i _at those same sites that specifically occurred in cathode-directed cells, not in anode-directed ones, during electrotaxis. However, intracellular levels of active NHE3 and of PIP2 were increased in anode-directed cells while they were less in cathode-directed cells in comparison to randomly migrating control cells. This can result from the localized activity of NHE3 in the membrane protrusions of cathode-directed cells. Most of the data concerning NHE3 deal with its renal functions; however, few studies have demonstrated the interactions between NHE3 and cytoskeletal proteins and the related signaling pathways regarding cell motility. The actin-modifying agents cytochalasin and latrunculin have been found to inhibit epithelial NHE3 activity, whereas the housekeeping exchanger, NHE1, has been found to be virtually affected [19]. Similarly, the cytoskeletal scaffolding protein ezrin has been shown to regulate NHE3 translocation in Caco-2 cells [20]. Consistent with these studies, we demonstrate significant colocalizations of active NHE3 with the filopodia marker protein ß-actin on the leading-edge of both anode- and cathode-directed cells. The distinct patchy pattern of NHE3 may depend on the activity of NHE3 because only active NHE3 shows this pattern and, this activity seems to be specialized in the filopodia where ß-actin is present. This might be due to the close interaction of these two proteins during cell migration. Additionally, the quenched directedness in the presence of the NHE3-specific inhibitor S3226 supports the idea that NHE3 is involved in directional determination during electrotaxis. Moreover, we have shown both a physiological and mechanistic role for NHE3 in cellular motility and directedness via demonstrating the colocalization of active NHE3 outward proton fluxes accompanied ß-actin (decreased pH_i_) at the leading edge of directionally migrating cells.

In contrast to NHE3, neither the expression nor the distribution of NHE1 was affected in directionally migrating cells during electrotaxis. Although the pharmacological inhibition of NHE1 via the use of HOE 642 prevented the cells from perceiving their direction which they normally would during electrotaxis, this phenomenon might be due to one of its other crucial functions as a housekeeping protein, e.g. the regulation of intracellular pH and volume, or to its roles in cell migration [21-25]. Therefore, we conclude that NHE1 is not specifically involved in determining the direction of migration, but instead, maintains the overall pH_i_, volume, and osmotic balance, which is crucial to cellular physiology during directed cell motility.

The relatively hyperpolarized cell membrane on the leading edge versus the rear end of the cells, regardless of their direction, can be explained by the relocation of NaKA from the cytoplasm to the leading edge in both cathode- and anode-directed cells during electrotaxis. NaKA is a major regulator of V_mem _and maintains the resting membrane potential by transporting two K^+ ^ions in and three Na^+ ^ions out of the cell. If the cell membrane is hyperpolarized on the leading edge, more NaKA needs to be recruited to those sites in order to reduce the excessive positive charges on the membrane and to bring V_mem _level down to its resting potential. NaKA has already been shown to be exponentially activated as a function of the V_mem_, which in turn leads to a hyperpolarized cell membrane [26,27]. Independent of its role in ion transport, evidence exists for the involvement of NaKA in PI3K signaling and cell motility [28], in epithelial polarization and the suppression of invasion [29], and for its interaction with actin [30]. Moreover, V_mem _itself has already been shown to induce cytoskeletal modifications in F-actin, microtubules, and vinculin [31] and, in adherens junctions [32] in endothelial cells. Our study demonstrates that NaKA colocalizes with the focal adhesion marker protein vinculin at the leading edge of both anode- and cathode-directed cells, and its inhibition by oubain quenches cellular directedness. This suggests that NaKA is involved in focal adhesion turnover, and, hence, the inhibition of its activity interferes with directional cell motility. Moreover, considering that the cell membrane is only hyperpolarized on the front and depolarized only on the back, regardless of the migration direction, V_mem _seems to maintain the persistent directedness rather than to perceive the direction by regulating the spatiotemporal NaKA mobility between the leading edge and the rear end of cells during directional cell motility. Additionally, both the total and phosphorylated NaKA levels were increased in cathode-directed (Calvaria) cells, which indicate that both the expression and the activity of the protein were elevated in those cells during electrotaxis. In contrast, in anode-directed (SaOS-2) cells, only the activity of the protein was increased, whereas the total level of the protein was not affected. These data indicate that NaKA activity is required in both anode- and cathode-directed cells.

Cellular speed was dramatically increased, especially in anode-directed cells, when NHE3 (300%) or NHE1 (500%) as well as FAK (500%, control) activity was inhibited. This suggests a motility suppressor role for both NHE3 and NHE1 in these cells during directional motility. An NHE inhibition-induced reduction in cell adhesion and motility has been reported in various cell types [33-35]. These studies state that the local extracellular pH levels at focal adhesion sites modulate the strength of the cell adhesion and thereby migration of the cells on a collagen I matrices. An increase in NHE1 activity (more protons) was also observed to result in tighter adhesion and decreased cell migration, whereas a lack of protons, due to low NHE1 activity, prevented adhesion and migration. Further studies should elucidate the exact mechanisms of interaction between NHE3 activity (pH_i_) and filopodia formation so as to understand its physio-mechanical roles in persistent, directional cell motility.

In contrast to NHE3 and NHE1, the pharmacological inhibition of NaKA using oubain divergently decreased and increased the speed of anode- and of cathode-directed cells by 71% and an increase by 67%, respectively. It has already been reported that independent of its role in ion transport, NaKA has various other functions in cells [36], including its role as a motility suppressor function in MDCK carcinoma cells [28]; however, the reason why NaKA activity divergently affects anode- and cathode-directed cells divergently in terms of cellular speed remains unknown and requires further investigation.

## Conclusions

**Figure 7:**
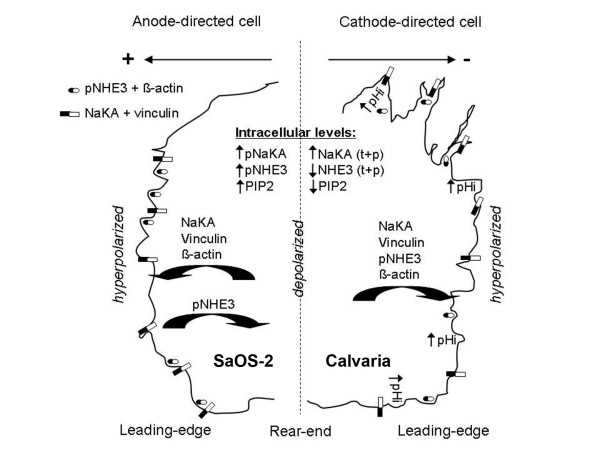
**A schematic of NaKA and NHE3 activity on the leading edge of cathode (Calvaria)- or anode (SaOS-2)-directed cells during electrotaxis**. Cells are polarized with a well-defined front and back during electrotaxis. Independent from the migration direction, NaKA is relocated from the cytoplasm to the protrusions, wherein its enhanced activity on the leading edges of cells and results in a hyperpolarized cell membrane, whereas the rear end membrane remains depolarized. Active NHE3 and consistently elevated pHi levels appear to be preferentially localized to the protrusions at the leading edges of cathode-directed cells, but not in anode-directed cells, which indicates a cathode-specific directional sensing. At the mechanical level, the colocalization of NaKA with vinculin at focal adhesion sites and the colocalization of active NHE3 with the filopodia marker ß-actin suggest a direct action for NaKA and for NHE3 in adhesion turnover and in filopodia formation, respectively.

Our study shows that NHE3, NHE1, and NaKA, in combination with spatiotemporal changes in V_mem _between the leading edge and the rear end, are required for the determination of cellular directedness during electrotaxis. Moreover, among these, NHE3 has unique responses that preferentially occur in cathode-directed cells, which suggests that it is a promising candidate for cathode-specific directional sensing (Figure 7). However, our interpretation would merit further testing, using V_mem _measurements through different tools to overcome the limitations of the technique used in this study. These data can be useful for further studies in which cell guidance is an important factor.

## Methods

### Osteoblast cell culture

Primary osteoblastic cells were isolated from fetal rat calvaria as previously described [37]. Cultures were maintained in DMEM/Ham's F12 media (Gibco BRL, Karlsruhe/Germany) that contained 12% fetal calf serum, 2.3 mM Mg^2+^, 100 μg/mL penicillin, 100 μg/ml streptomycin sulfate, and 1.25% L-glutamine at 37°C and in humidified 5% CO_2_. Between five and seven passages were taken for the experiments. Human osteosarcoma cells (SaOS-2), which are non-transformed cells with osteoblastic properties, were obtained from the American Type Culture Collection (ATCC HTB 85) and cultured in a McCoy's 5A media (Gibco BRL, Karlsruhe/Germany) that contained 15% fetal calf serum and 1.25% L-glutamine. SaOS-2 cells were used until passage number 50 because they are suspected to loose their osteoblast-phenotypic features in later passages [38]. For EF applications, 100 μL of cells at a concentration of 20,000 cells/mL were seeded into the channel of an ibiTreat μ-Slide I (Ibidi, Munich/Germany).

### Electric field stimulation

Direct current which was provided by an electrophoresis power supply (BioRad, Munich/Germany), was applied to the cells through two platinum electrodes (0.2 mm in diameter, Agar scientific, Essex/UK), which were immersed in 0.9% NaCl in water-filled beakers that were connected to the media-filled reservoirs of the μ-Slide by two 20-cm- long agar bridges (2% agar in PBS). HBSS and cell culture media was used for the ion imaging and migration assays, respectively. Agar bridges were used to avoid contact between the electrode products and the cells [39]. During the experiment, field strengths were measured by a voltmeter (Voltcraft^® ^Meßtechnik, Hirschau/Germany). The cells were exposed to a physiological range dcEF strength of 0.5 V/mm for 5 h at 37°C in a chamber (Solent Scientific, Segensworth/UK) that covered the entire inverted microscope or in an the incubator. The same conditions were used for controls but without the application of a dcEF.

### Cell motility assay

Cells were monitored via an Olympus IX81 inverted microscope that was equipped with DIC components and an integrated vital microscopy chamber (Olympus, Hamburg/Germany). An average number of 40 cells were scored for each condition. The cells were kept in their usual culture medium and exposed to EF with or without the presence of the inhibitors. Both the NHE3- and NHE1-specific inhibitors, S3226 and HOE 642, were provided by Sanofi-Aventis (Frankfurt/Germany). The NaKA inhibitor, ouabain octahydrate, was purchased from Sigma Aldrich (Munich/Germany). The FAK inhibitor, PF573228, was purchased from Tocris Biosciences (Bristol/UK).

All of the collected data were analyzed with the Olympus imaging software Cell^R. In order to quantify the cell speed (μm/h) and net linear displacement (μm), time-lapse DIC videos were processed in the detecting program, TrackIT. Polar diagrams that were indicative of directedness were created using PSI-Plot (Poly Software International, New York/USA). Briefly, dots shown in the polar diagrams are corresponding to the objects (cells) whose movement has been tracked during the time course of migration. The software (TrackIT, Olympus) we used here to analyze the cell motility displays the data on cellular speed, net linear displacement and directedness separately in the graphical form. All these data series were then converted into an excel sheet using this program. Polar diagrams are widely used for presenting the data on cellular directedness in the motility assays. Polar diagrams we show in this study has been created using PSI Plot software (Poly Software International). The cellular directedness (position of the dots within 360°) in each of those diagrams is relative to the directedness of the control cells without EF; X axis having the data series analyzed from the control cells without EF and Y axis having those from the cells that exposed to only EF or EF + inhibitor. Graphics that depicted the speed and distance were created in Excel. A 10 X objective was used for visualizing the cells during the cell motility assay.

### Quantitative immunocytochemistry

Cells were washed once with PBS (pH 7.4), fixed in 4% formaldehyde/0.05% glutaraldehyde for 5 min at room temperature (RT), permeabilized with 10 μg/ml digitonin (Sigma Aldrich, Munich/Germany) or with 0.5% Triton X-100 for 6 min (Sharma et al., 2008), and then blocked with 1% BSA for 20 min. Cells were then incubated with a primary antibody overnight at 4°C. After washing with PBS, cells were further treated with fluorescence-coupled antibodies (FITC/TexasRed) for 1 h at RT. Dabco-glycerin in PBS was used as a mounting solution. The sources of the antibodies included mouse monoclonal anti-Na^+^/K^+^-ATPase (α3 subunit, 1:200, Sigma Aldrich, Munich/Germany); goat polyclonal anti-phospho Na^+^/K^+^-ATPase (α-Ser 943, 1:100, Santa Cruz Biotechnology Inc., Heidelberg/Germany); mouse monoclonal anti-NHE-3 isoform (1:50, BD Transduction Labs, Heidelberg/Germany); mouse monoclonal anti-phospho NHE-3 (Ser 552, 1:500, Novus Biologicals, Heford/Germany); rabbit anti-human vinculin (1:200, Sigma Aldrich, Munich/Germany); mouse monoclonal anti-phosphatidylinositol 4,5-bisphosphate (PIP2, 1:200, Abcam, Cambridge/UK); rabbit polyclonal anti-beta actin (1:1000, Novus Biologicals, Heford/Germany); fluorescein-isothiocyanate (FITC)-coupled anti-mouse, anti-rabbit, or anti-goat (1:500, Dianova, Hamburg/Germany); Texas Red-coupled anti-mouse and anti-rabbit (1:500, Dianova, Hamburg/Germany). A 40 X objective was used for visualizing the cells. Image J was used to analyze the colocalization rate. For this, red:green combination of the channel color was set for FITC and Texas red, respectively. Then, regions of interests (ROIs) were placed where the colocalizations are observed to analyze the pixels in the defined color channels within the ROI selected. Results window displayed automatically show Pearson's correlation coefficient whose values range from 1 to -1, where "1" indicates a perfect correlation. The plots showing the overlap rates of the proteins are generated in Excel based on their Pearson's correlation coefficient values. Relocation rate was quantified using Cell^R (Olympus) software. For quantification of relocation, three ROIs were inserted in the cytoplasm and the membrane protrusions. The mean fluorescence intensity (MFI) profiles were calculated from the quotient of the ROIs at the membrane protrusions and the ROIs in the cytoplasm. The following formula was used to calculate the relocation rate:

(1) $$Relocation\;Rate=\frac{\sum MFI\;ROIs\;lamellopodia}{\sum MFI\;ROIs\;cytoplasm}$$

### FACS measurements

Protein expression levels were analyzed by using fluorescence activated cell sorter (FACS Calibur, BD GmbH, Heidelberg/Germany). Control and EF-exposed cells were trypsinized from the Ibidi μ-slides and then separately collected into the FACS tubes. Cells were washed once with DPBS (pH 7.4), fixed in 4% formaldehyde/0.05% glutaraldehyde at room temperature (RT) for 10 min and then centrifuged at 1200 rpm for 4 min. Next, the cells were permeabilized with 10 μg/ml digitonin for 10 minutes, centrifuged, and then blocked with 0.5% BSA for 30 min. The cells were then incubated with a primary antibody for 1 h at RT. After washing with DPBS, the cells were then treated with FITC-conjugated antibodies for 1 h at RT. Finally, the cells were washed with DPBS and FACS was used to measure their relative fluorescence intensity. For each analysis, 5,000 cells were counted. For the detection of proteins of interest, different sources of antibodies were used, including mouse monoclonal anti-Na^+^/K^+^-ATPase (α3 subunit, 1:100, Sigma Aldrich, Munich/Germany); goat polyclonal anti-phospho Na^+^/K^+^-ATPase (α-Ser 943, 1:100, Santa Cruz Biotechnology Inc., Heidelberg/Germany); mouse monoclonal anti-Na^+^/H^+^exchanger (NHE)-1 isoform (1:50, BD Transduction Labs, Heidelberg/Germany); mouse monoclonal anti-NHE-3 isoform (1:50, BD Transduction Labs, Heidelberg/Germany); mouse monoclonal anti-phospho NHE-3 (Ser 552, 1:200, Novus Biologicals, Heford/Germany); mouse monoclonal anti-phosphatidylinositol 4,5-bisphosphate (PIP2, 1:100, Abcam, Cambridge/UK) and fluorescein-isothiocyanate (FITC)-coupled anti-mouse and anti goat (1:100, Dianova, Hamburg/Germany).

### Immunoprecipitation

Cells were washed with ice-cold PBS. Whole cell lysates were prepared with a RadioImmunoPrecipitation Assay (RIPA) buffer by gentle scraping. The RIPA buffer was prepared with 50 mM Tris HCL at a pH of 8, 150 mM NaCl, 1% NP-40, and 0.5% sodium deoxycholate. Pre-clear lysates were incubated with either mouse monoclonal anti-Na^+^/K^+^-ATPase (α3 subunit, 1:500, Sigma Aldrich, Munich/Germany) or mouse monoclonal anti-Na^+^/H^+^exchanger (NHE)-1 isoform (1:250, BD Transduction Labs, Heidelberg/Germany), or mouse monoclonal anti-phospho NHE-3 (Ser 552, 1:250, Novus Biologicals, Heford/Germany) for 1 h at RT. These lysates were further incubated with immobilized protein A/G beads (ImmunoPure, Pierce) for 2 h at 4°C. Finally, these beads were washed three times with a RIPA buffer and centrifuged at 600 g for 5 min.

### Western Blot

Proteins were eluted from the beads via the addition of 50 μL of a 2x SDS-PAGE sample buffer. After heating at 95°C for 5 min, proteins were separated based on their size by an SDS that contained (10%) polyacrylamide (Roth, Karlsruhe/Germany) gels and then transferred to PVDF membranes (Millipore, Schwalbach/Germany) by the semi-dry transfer method. Membranes were blocked at RT for 30 min with TBST (TBS with 0.1% Tween-20), which contained 5% skim milk powder. Blots were then incubated overnight at 4°C with primary antibodies, including mouse monoclonal anti-Na^+^/K^+^-ATPase (α3 subunit, 1:500, Sigma Aldrich, Munich/Germany), mouse monoclonal anti-Na^+^/H^+^exchanger (NHE)-1 isoform (1:250, BD Transduction Labs, Heidelberg/Germany), or mouse monoclonal anti-phospho NHE-3 (Ser 552, 1:250, Novus Biologicals, Heford/Germany). Blots were washed three times (5 min each) with TBST. For standard Western blot detection, the blots were incubated for 1 h at RT with an anti-mouse HRP conjugated antibody (1:2000, Amersham Biosciences, Freiburg/Germany). Afterwards, the blots were developed with chemiluminescent HRP substrate (Immobilon Western, Millipore, Schwalbach/Germany) using image reader-LAS 3000 (Fujifilm, Dusseldorf/Germany).

### The real-time imaging of pH and the cell membrane potential

Ion-specific vital dyes were used to monitor dcEF induced changes in intracellular ions and in cell membrane potential. Ratiometric dye BCECF-AM was used as a reporter dye for intracellular pH (pH_i_) and potentiometric dye DiBAC_4_(3) was used for cell membrane potential (V_mem_). DiBAC4(3) has already been successfully used to assess the differences in the membrane potential both in vivo [40,41] and in vitro [42,43] - studies. Both dyes were purchased from Invitrogen (Darmstadt/Germany). After 5 h of EF exposure, the cells were washed once with HBSS and then loaded with 0.5 μM DiBAC_4_(3) in HBSS at 37°C for 30 min. Subsequently, V_mem _dye uptake and intracellular localization was monitored using an inverted microscope with the appropriate filters for the DiBAC_4_(3) dye (excitation/emission; 493 nm/516 nm). V_mem _kinetics were generated from the selected region of interests during the time-lapse recording of the cells loaded with DiBAC_4_(3). A background region was also included in the settings during time-lapse recordings for background subtraction. For pH_i _monitoring, the cells were loaded with 1 μM BCECF-AM dye in HBSS and merged images generated from the dual excitation (490 nm and 440 nm, emission; 530 nm) were recorded using an inverted microscope with the specific filters for BCECF-AM. pH bubbles were observed at the leading edge of the cells when the cells were exposed to further stimulation with 0.5 V/mm EF. The cells were monitored using the imaging software Cell^R and a 40X objective.

### Statistics

Statistical calculations were performed in PSI-Plot using paired or unpaired Student's t-tests, or ANOVA. A *P *< 0.05 was defined as significant (* or §). Data are presented as mean ± SEM.

## Authors' contributions

SP and NÖ carried out the immnunocytochemical, FACS, cell motility and time-lapse video recording studies. PS is carried out the inhibition and western blot studies. NÖ, SP and PS analyzed the data. NÖ performed the statistical analysis. NÖ and SP drafted and wrote the manuscript. NÖ and RHWF conceived, designed and coordinated the study. All authors read and approved the final manuscript.

## Supplementary Material

Additional file 1 **Intracellular distribution of NHE3 (TRITC-labeled)**. The total NHE3 is evenly distributed with slight accumulations in the membrane protrusions in both polarized (+EF, right panel) and non-polarized (-EF, left panel) cells during cathode (Calvaria)- or anode (SaOS-2)-directed motility. Click here for file

Additional file 2 **Intracellular distribution of NHE1 (FITC-labeled)**. The cellular distribution of NHE1 (left panel) is not affected during cathode (Calvaria)- or anode (SaOS-2)-directed motility (right panel). Click here for file

Additional file 3 **Intracellular distribution of phosphorylated NaKA (FITC-labeled)**. Phosphorylated NaKA is homogenously distributed at the cell membrane during directed motility in both cathode (Calvaria)- or anode (SaOS-2)-directed cells. Click here for file

Additional file 4 **Intracellular distribution of PIP2 (FITC-labeled)**. PIP2 localizes along the cell periphery both in anode (SaOS-2) and cathode-directed (Calvaria) cells. Click here for file

Additional file 5 **Time lapse imaging of DiBAC(4)3 uptake into the cathode-directed (Calvaria) cells**. The dye enters into the cells through the depolarized membrane at the rear end of the polarized cells regardless of their direction. Images were collected every 35 sec for 20 min using inverted fluorescence microscopy. Click here for file

Additional file 6 **Time lapse imaging of DiBAC(4)3 uptake into the anode-directed (SaOS-2) cells**. The dye enters into the cells through the depolarized membrane at the rear end of the cells regardless of their direction. Images were collected every 35 sec for 20 min using inverted fluorescence microscopy. Click here for file

Additional file 7 **Time lapse imaging of H^+ ^fluxes at the cell periphery**. pH bubble formation at the termini of membrane protrusions on the leading-edge of cathode-directed (Calvaria) cells during electrotaxis. Cells were loaded with BCECF-AM, a pH reporter, ratiometric dye. Images were collected every 9 sec for 25 min using inverted fluorescence microscopy. Click here for file
