# Supplementary figures and images for: Persistent directional cell migration requires ion transport proteins as direction sensors and membrane potential differences in order to maintain directedness
Source: BMC Cell Biol. 2011 Jan 22;12:4. doi: 10.1186/1471-2121-12-4 (PMC3042415; doi:10.1186/1471-2121-12-4)

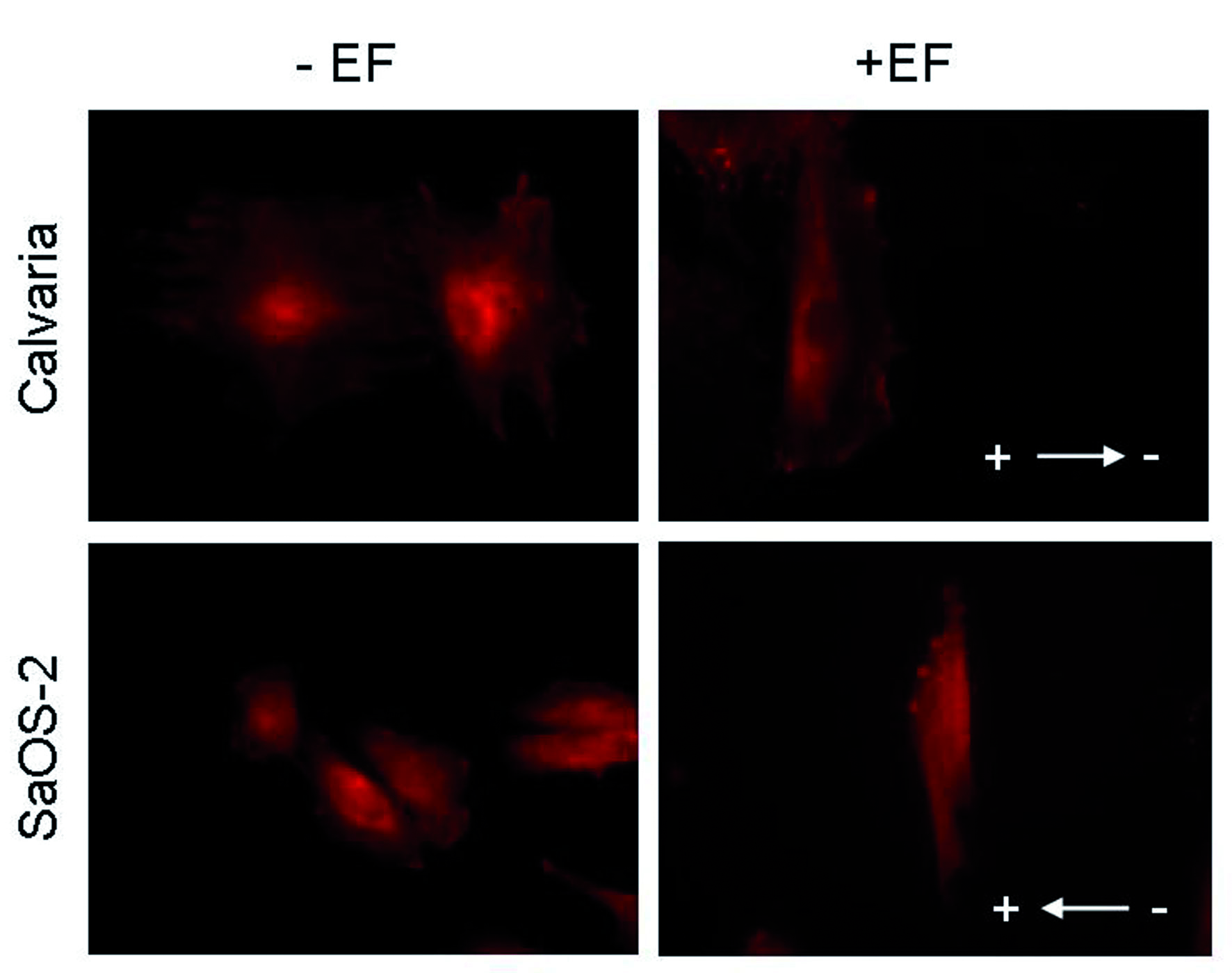

Supplement: Additional file 1 — Intracellular distribution of NHE3 (TRITC-labeled). The total NHE3 is evenly distributed with slight accumulations in the membrane protrusions in both polarized (+EF, right panel) and non-polarized (-EF, left panel) cells during cathode (Calvaria)- or anode (SaOS-2)-directed motility. [file 1471-2121-12-4-S1.JPEG]

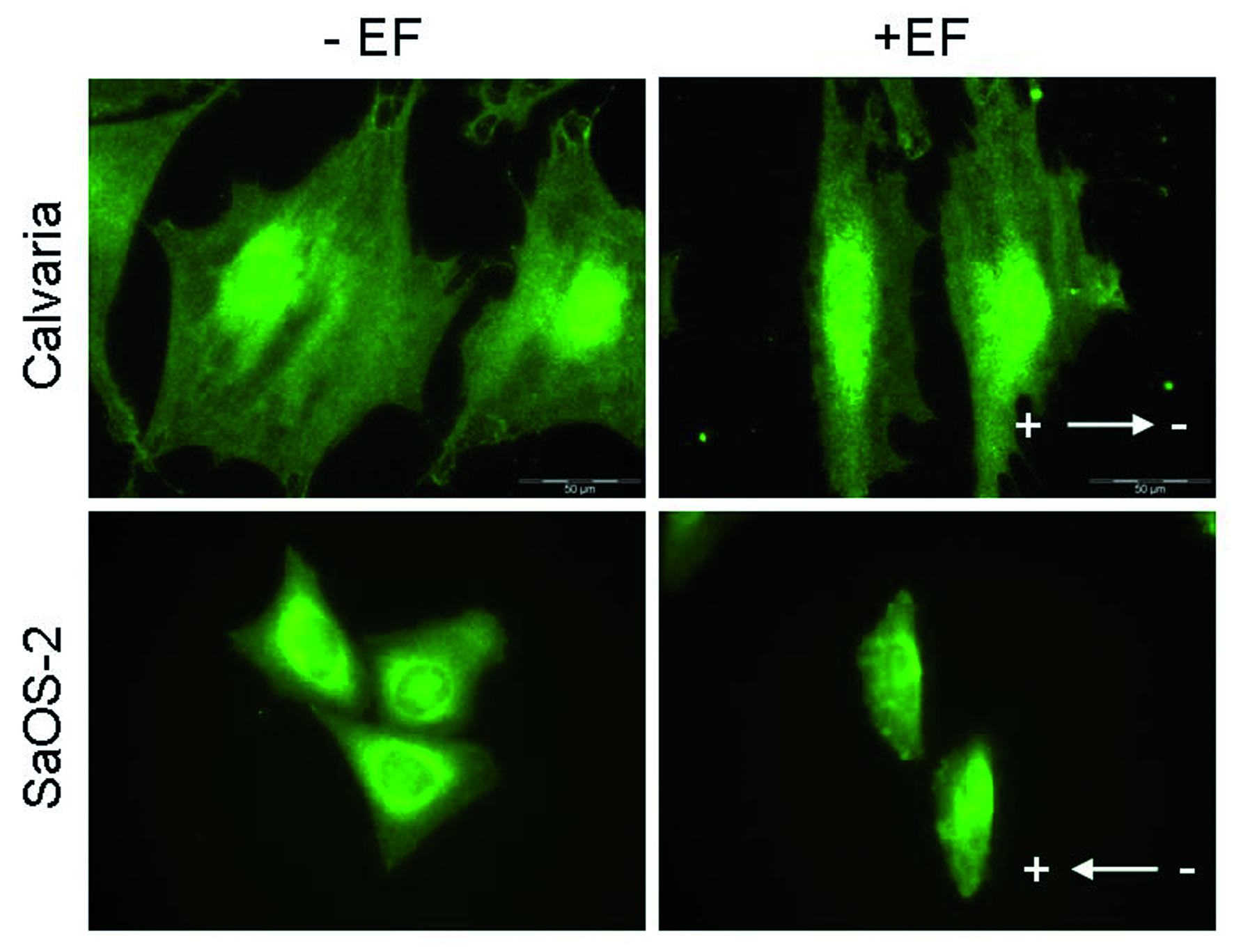

Supplement: Additional file 2 — Intracellular distribution of NHE1 (FITC-labeled). The cellular distribution of NHE1 (left panel) is not affected during cathode (Calvaria)- or anode (SaOS-2)-directed motility (right panel). [file 1471-2121-12-4-S2.JPEG]

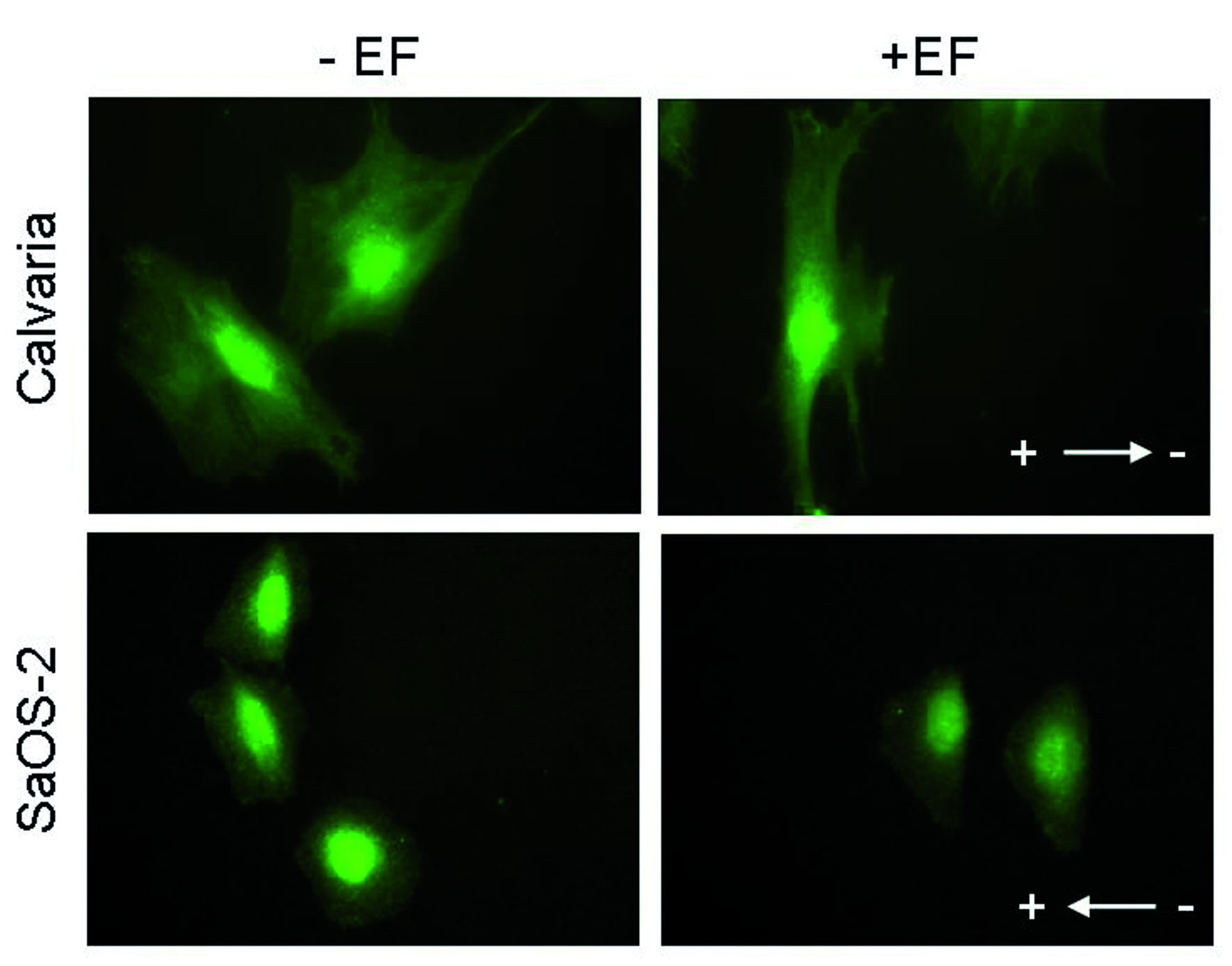

Supplement: Additional file 3 — Intracellular distribution of phosphorylated NaKA (FITC-labeled). Phosphorylated NaKA is homogenously distributed at the cell membrane during directed motility in both cathode (Calvaria)- or anode (SaOS-2)-directed cells. [file 1471-2121-12-4-S3.JPEG]

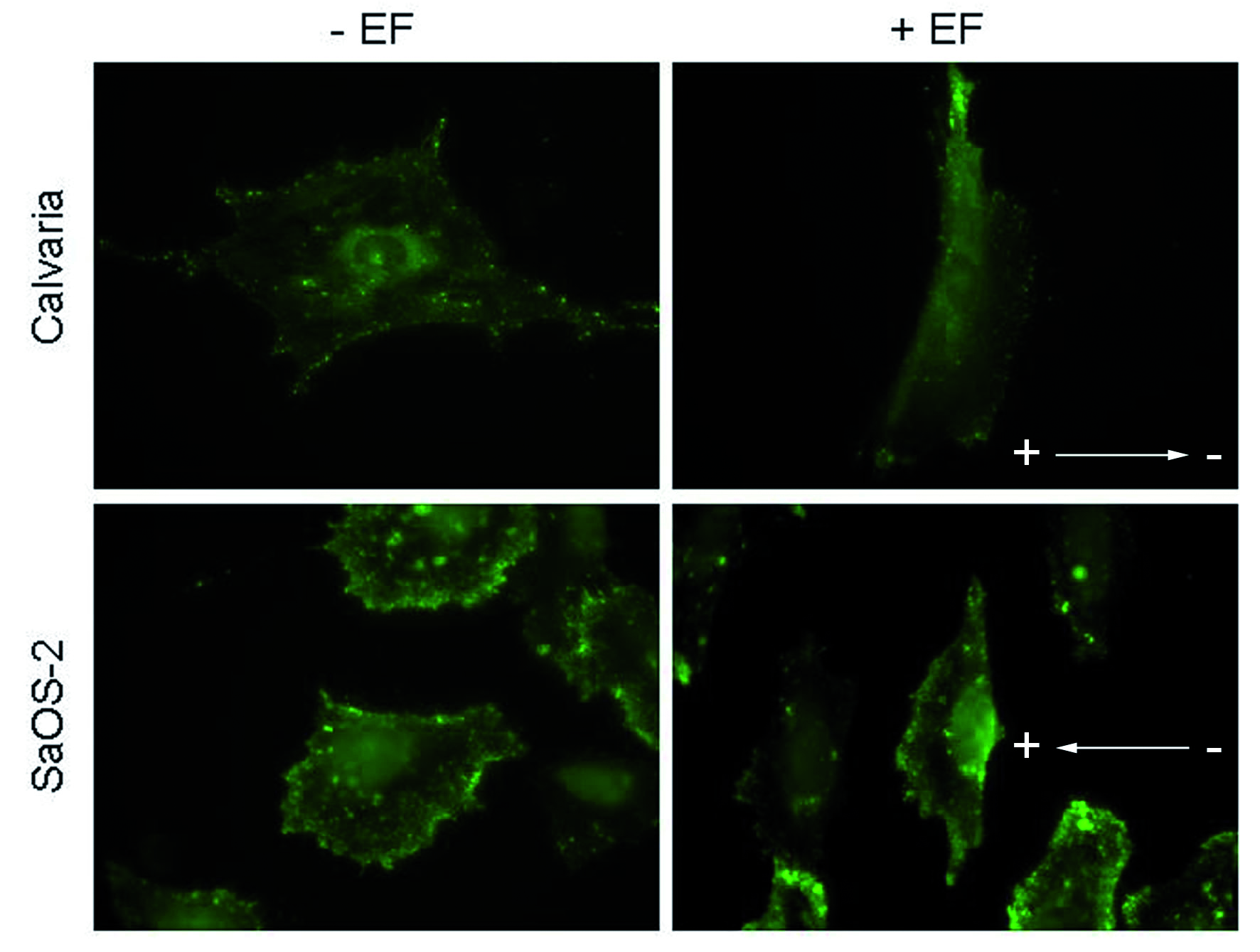

Supplement: Additional file 4 — Intracellular distribution of PIP2 (FITC-labeled). PIP2 localizes along the cell periphery both in anode (SaOS-2) and cathode-directed (Calvaria) cells. [file 1471-2121-12-4-S4.JPEG]
